# An atomic resolution description of folic acid using solid state NMR measurements

**DOI:** 10.1039/d0ra03772a

**Published:** 2020-07-01

**Authors:** Manasi Ghosh, Shovanlal Gayen, Krishna Kishor Dey

**Affiliations:** Department of Physics, Dr Harisingh Gour Central University Sagar-470003 MP India manasi.ghosh@gmail.com dey.12@buckeyemail.osu.edu; Department of Pharmaceutical Sciences, Dr Harisingh Gour Central University Sagar-470003 MP India

## Abstract

The chemical shift anisotropy tensor and site-specific spin-lattice relaxation time of folic acid were determined by a ^13^C 2DPASS CP-MAS NMR experiment and Torchia CP experiment respectively. The molecular correlation time at various carbon nuclei sites of folic acid was evaluated by assuming that the ^13^C spin-lattice relaxation mechanism is mainly governed by chemical shift anisotropy interaction and hetero-nuclear dipole–dipole coupling. CSA parameters are larger for the carbon nuclei residing at the heteroaromatic ring and aromatic ring, and those attached to double-bonded electronegative oxygen atoms. It is comparatively low for C9, C19, C21, and C22. The molecular correlation time is of the order of 10^−4^/10^−5^ s for C9, C19, C21 and C22 carbon nuclei, whereas it is of the order of 10^−3^ s for the rest of the carbon nuclei sites. Spin lattice relaxation time varies from 416 s to 816 s. For C23 and C14, the value is 816 s, and it is 416 s for C7 nuclei. The correlation between structure and dynamics on an atomic scale of such an important drug as folic acid can be visualized by these types of extensive spectroscopic measurements, which will help to develop an advanced drug for DNA replication.

## Abstract 2

The chemical shift anisotropy tensor and site specific spin-lattice relaxation time of folic acid were determined by a ^13^C 2DPASS CP-MAS NMR experiment and Torchia CP experiment respectively.

## Graphical abstract

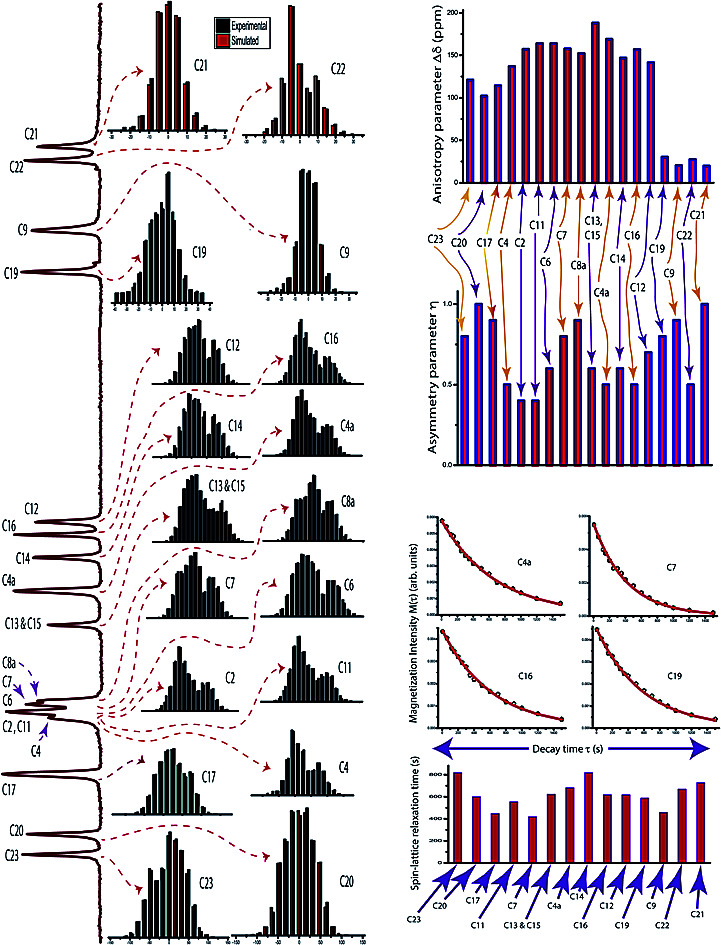

## 1. Introduction

Folic acid (vitamin B9) is indispensable for DNA replication, growth, and development of the fetus. This synthetic form of folate is used as a substrate for enzymatic reactions associated with amino acid synthesis and vitamin metabolism. Deficiency of folic acid can cause congenital abnormalities, anemia, and peripheral neuropathy. The body cannot synthesize folic acid; it can be obtained from diet or supplementation. Dietary supplement of folic acid during pregnancy decreases the hazard of neural tube defects (NTD) in the offspring, reduces the risk of preterm birth (PTB) and protects against inborn heart defects.^1–5^ Folic acid and its derivatives play a vital role in the process of formation of purine and thymidine, which are considered as essential ingredients for the formation of nucleic acids. Folic acid also plays a pivotal role in the synthesis of the methylation agent *S*-adenosyl-methionine, which is essential for protein and lipid production.^4^

Nuclear magnetic resonance (NMR) spectroscopic technique plays a central role to elucidate the three-dimensional structure and dynamics of a molecule with atomic resolution at sufficient sensitivity. NMR interactions can be classified into two categories-spin-field interactions and spin–spin interaction. Zeeman, chemical shift, magnetic susceptibility, and radio-frequency field interactions are labeled as spin-field interactions. Whereas homo-nuclear dipole–dipole coupling, hetero-nuclear dipole–dipole coupling, J-coupling, and quadrupole coupling are designated as spin–spin interactions. Although at high value of the magnetic field, the strongest interaction is the Zeeman interaction, it failed to provide any direct information about the three-dimensional structure and spin dynamics of a molecule, because its value is the same for all the nuclei of the same kind. The effective magnetic field experienced by the nucleus or the Larmor precession frequency of the nucleus is modified due to the presence of the electron cloud surrounding the nucleus – this is referred as nuclear shielding or de-shielding effect and the change in the nuclear resonance frequency due to this effect is known as chemical shift. The electronic distribution around a nucleus has low-symmetry. Hence, the chemical shift has an anisotropic part as well as an isotropic part. The anisotropic part depends on the molecular conformation and orientation of the molecule with respect to the external magnetic field. Chemical shift anisotropy can be represented mathematically by a second-rank tensor associated with nine components. In principal axis system (PAS) only the diagonal components restored. The expressions of those diagonal components of the chemical shift anisotropy tensor (*δ*_11_, *δ*_22_ and *δ*_33_) are given by^6,7^
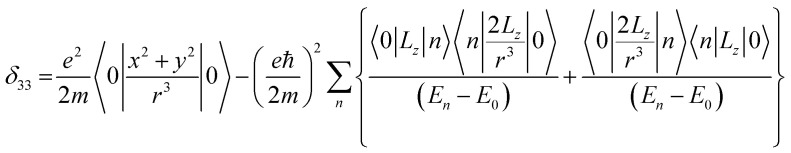

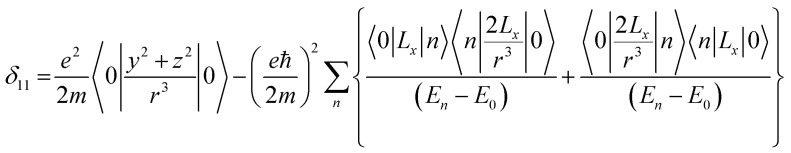

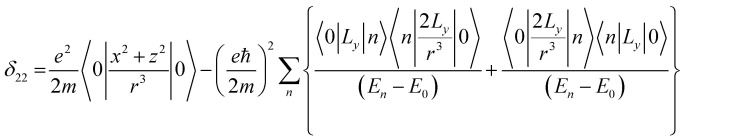
where *L*_*x*_, *L*_*y*_ and *L*_*z*_ are the components of angular momentum along the *x*, *y* and *z* directions respectively. ‘*r*’ is the position of the electron with respect to the nucleus. The first part of these three equations arises from spherically symmetric electronic charge distribution *i.e.* when the electrons reside in the ground state (the ‘s’ orbital state). The second term arises due to the distortions in the symmetry of the charge distribution when electrons are in excited states. *δ*_11_ corresponds to minimum nuclear shielding with maximum nuclear resonance frequency. Conversely, *δ*_33_ corresponds to maximum nuclear shielding with minimum nuclear resonance frequency molecule has no preferred direction of orientation for liquid sample, it can orient in any direction. Hence, the averaging of CSA tensor over all possible directions of orientation generates the isotropic chemical shift *δ*_iso_ = (*δ*_11_ + *δ*_22_ + *δ*_33_)/3. Hence, for solid sample it is possible to extract information about the electronic distribution surrounding the nucleus by determining the values of *δ*_11_, *δ*_22_, and *δ*_33_.

There are several techniques to determine CSA parameters – two dimensional MAS/CSA NMR experiment;^8^ SUPER (separation of undistorted powder patterns by effortless recoupling);^9^ ROCSA (recoupling of chemical shift anisotropy);^10^ RNCSA(γ-encoded RN_*n*_^*ν*^-symmetry based chemical shift anisotropy);^11^ 2DMAF (two-dimensional magic angle flipping) experiment;^12–15^ 2DMAT (two-dimensional magic angle turning) experiment;^16^ and two-dimensional phase adjusted spinning side-band (2DPASS) cross-polarization(CP) magic angle spinning (MAS) SSNMR experiment.^17,18^ 2DPASS CP-MAS SSNMR technique was applied to elucidate the structure and dynamics of glass, biopolymers, biomedicine.^19–25^

5,6,7,8-Tetrahydrofolate is an important cofactor for the synthesis of thymidylate from deoxyuridylate. If the body fails to synthesize thymidylate, DNA synthesis and cell division would not be possible. Hence, blockade of tetrahydrofolate formation eventually leads to cell death. Folic acid reduces to 7,8-dihydrofolate and tetrahydrofolate step by step within the human body by the action of the enzyme dihydrofolate reductase (DHFR).^26^ This is a fascinating system for developing anticancer agents. It is necessary to have in-depth information about the structure and dynamic of folic acid. The aim of this work is to study the structure and dynamics of this essential dietary supplement by extracting chemical shift anisotropy (CSA) parameters by 2DPASS CP-MAS NMR experiment; spin-lattice relaxation time at chemically different carbon nuclei positions by Torchia CP method; and determination of molecular correlation time at numerous carbon nuclei position. This type of research will enlighten the path of inventing dietary supplements, which can reduce the risk of birth defects.

## 2. Experimental

### 2.1 NMR measurements

Folic acid, purchased from Sigma Aldrich, was used for the NMR experiment. The purity of the sample was 99.98%. NMR experiments were performed on a JEOL ECX 500 NMR spectrometer. Nearly 50 mg folic acid was tightly packed in 3.2 mm JEOL double resonance MAS probe for performing solid state NMR experiments. ^13^C CP-MAS and ^15^N CP-MAS experiments were done at magic angle spinning speed 10 kHz. The CP contact time was 2 ms to satisfy the state of cross-polarization (CP). SPINAL-64 ^1^H decoupling was used during acquisition. External referencing for ^15^N was done by NH_4_Cl. ^13^C spin-lattice relaxation time was measured by the Torchia CP method.^35^ The number of scans for ^13^C CP MAS experiment scan was 32 768 and for ^13^C 2DPASS CP MAS experiment number of scans was 4030. For measuring ^13^C relaxation by the Torchia CP method, the number of scans was 2048.

### 2.2 CSA measurements

Chemical shift anisotropy parameters of folic acid were evaluated by 2DPASS CP MAS SSNMR experiment.^17,18^ Thirteen steps cogwheel phase cycling was used. Sixteen data points were acquired in the indirect dimension as the numbers of sidebands were less than sixteen. π pulse spacing was given in Tabular form by Antzutkin *et al.*^18^ 90° pulse length for ^13^C was 3.3 us. The Relaxation delay was 10 s. The number of scans for the experiment were 4030 (integral multiple of 13). The coherence transfer pathway diagram for 2D PASS NMR experiment was given in Ghosh *et al.*^20^ With the help of this experiment, the isotropic dimension can be correlated with anisotropic dimension by shearing transformation and two dimensional Fourier Transformation. The overlapping signals can be identified by the 2DPASS CP MAS SSNMR experiment, as their CSA parameters are different. CSA eigenvalues can be determined by using the sideband intensities of spinning CSA sideband pattern of chemically different carbon nuclei.^27^ 2DPASS CP MAS SSNMR experiment is associated with five π pulses.^18^ The time evolution of theses five π pulses is varied according to the PASS equation, but the total time duration of all the PASS sequences are same. 2DPASS experiments were performed at MAS speed 600 Hz and 2 kHz.

## 3. Results and discussion

### 3.1 NMR spectral analysis

**Fig. 1:**
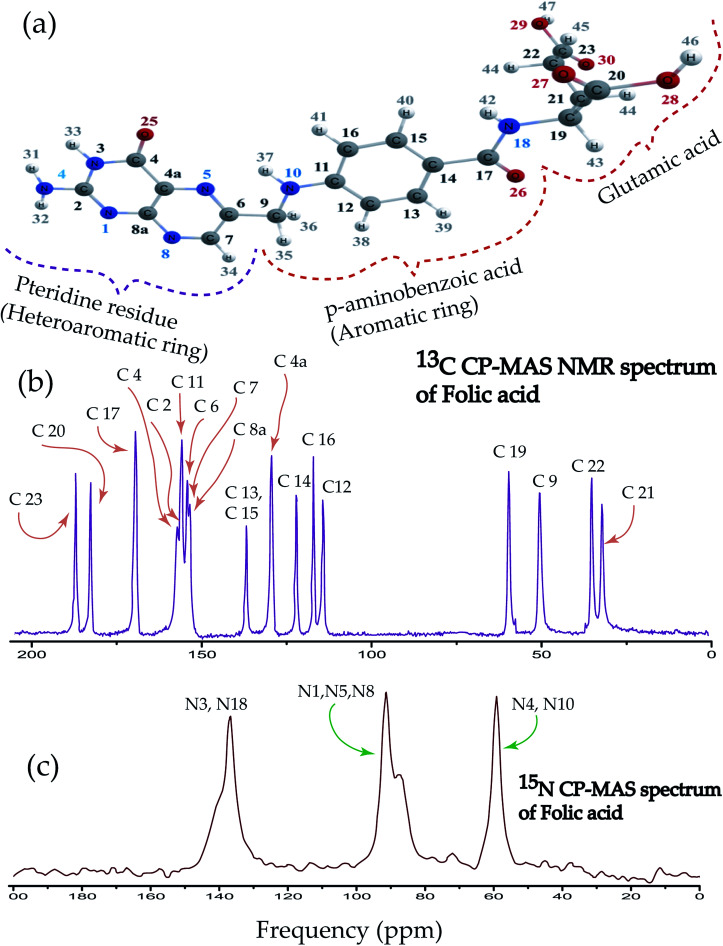
(a) Folic acid is fabricated by 2*S*-2-{[4-(2-amino-4hydroxypteridin-6-yl)-methyl]amino}phenyl formamido pentanedioic acid. It is composed of pteridine, *p*-aminobenzolate and glutamate moieties. (b) ^13^C CP-MAS spectrum of folic acid. (c) ^15^N CP-MAS spectrum of folic acid.

Folic acid is 2*S*-2-{[4-(2-amino-4hydroxypteridin-6-yl)-methyl]amino}phenyl formamido pentanedioic acid. Fig. 1(a) shows that the folic acid molecule is composed of pteridine, *p*-aminobenzolate, and glutamate moieties. Aminobenzoyl moiety acts as a connector between pteridine and glutamate moieties and it is strongly bound to the receptor. Hence, it acts like ‘anchors’ between the ligand and the receptor. Glutamate moiety is formed by two isonizable carboxylic acid functional groups, which can be used to append with other molecules or to constitute salt-bridges with receptor residues.^4^ The biologically inactive folic acid becomes biologically active within the liver by forming dihydrofolic and terahydrofolic acids. Fig. 1(b) shows ^13^C CP-MAS NMR spectrum of folic acid. The isotropic chemical shift is very high for C23, C20, C17, C4 carbon atom associated with double bonded oxygen, and it is lowest for C21, C22, C9, and C19. Chemical shift is highly correlated with the presence of electronegative atoms in the neighbourhood. Electronegative atom attracts the electron cloud around the carbon nucleus. As a consequence, the electron cloud density is reduced and the effective field experienced by the carbon nuclei is increased, leads to the higher values of chemical shift. The nucleus C9 resides on the symmetry axis of a rigid molecule with a strong magnetic susceptibility experienced the magnetic field, which is lower than the applied magnetic field – shielding effect. On the contrary, the local magnetic field experienced by the nuclei C4a, C8a, C6, C7 reside in the plane of the rigid molecule is higher than the applied magnetic field – known as deshielding effect. This phenomenon is also known as the ring-current shift. In the next section, it will be discussed that the chemical shift anisotropy (CSA) parameters are also very high for C4a, C8a, C6, C7 compared to C9 nucleus. Three well resolved resonance peaks are observed in ^15^N CP-MAS NMR spectrum of folic acid (Fig. 1(c)). The overlapping resonance line near 140 ppm originates from N3 and N18 nuclei. The resonance line near 90 ppm comes from N1, N5, N8 nuclei resides on pteridine ring. Resonance line near 60 ppm comes from N4 and N10 nuclei.

### 3.2 Chemical shift anisotropy parameters

**Fig. 2:**
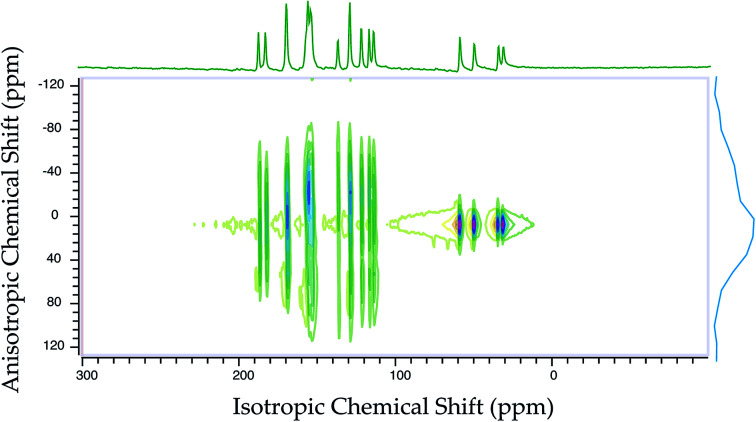
^13^C 2DPASS CP-MAS NMR spectrum of folic acid. The direct dimension corresponds to the isotropic spectrum with zero sideband, which is known as the infinite spinning speed spectrum. The indirect dimension corresponds to the anisotropic spectrum.

**Fig. 3:**
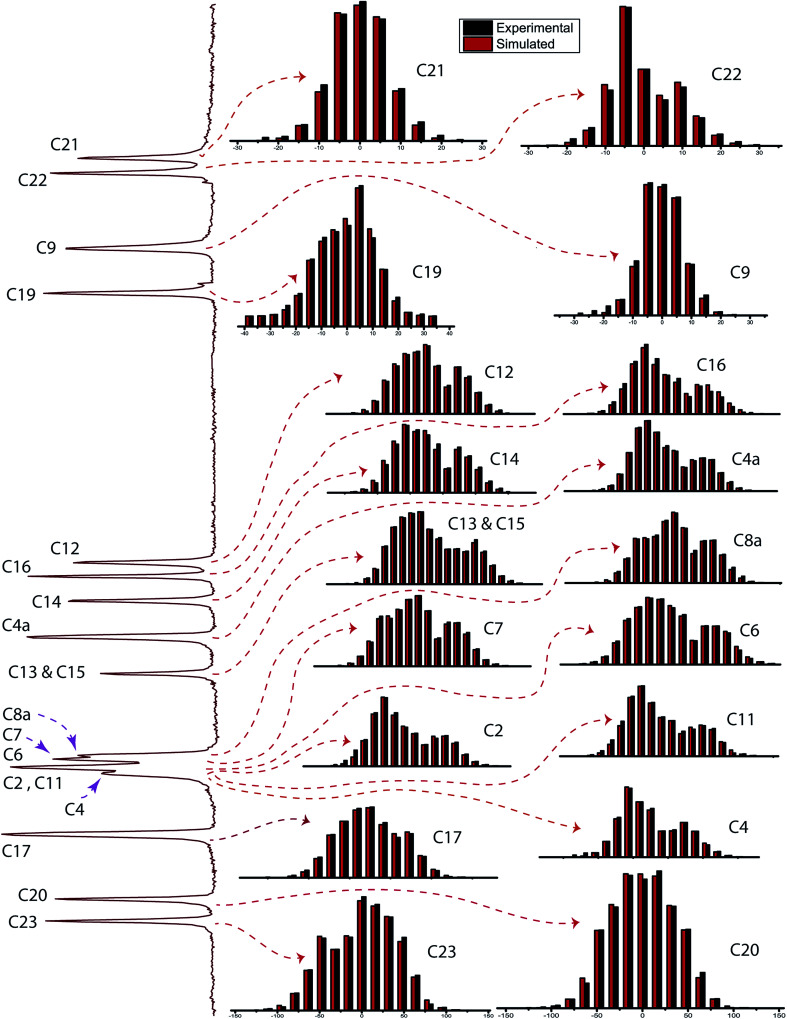
Spinning CSA sideband pattern corresponding to numerous carbon nuclei with chemically different atmosphere is shown in figure. The span (*Ω* = *δ*_11_ − *δ*_33_) of C9, C19, C21, C22 is much lower than that of the other carbon nuclei. The spinning CSA sideband pattern corresponding to C9, C19, C21, C22 carbon nuclei are obtained by 2DPASS CP MAS NMR experiment at spinning speed 600 Hz, where the rest of the sideband patterns are obtained at MAS speed 2 kHz.

**Fig. 4:**
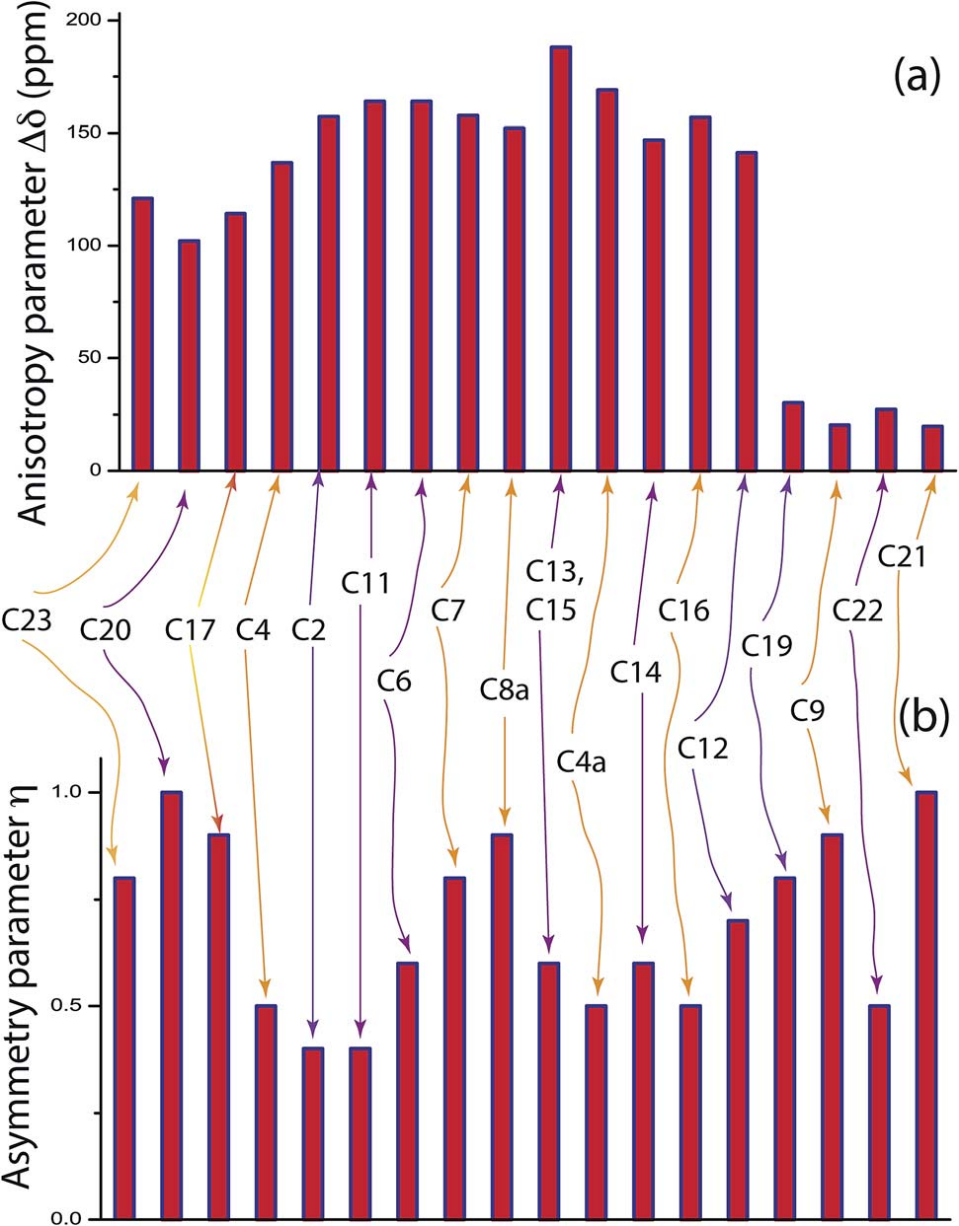
(a) Anisotropy parameter 
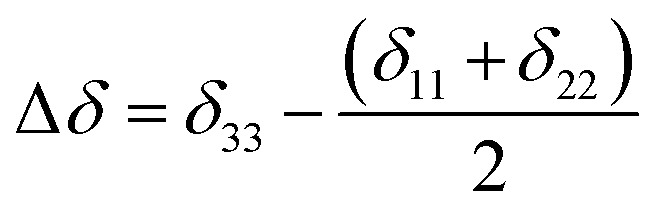
 for crystallographically different carbon site of folic acid. The magnitude of anisotropy parameter is remarkably low for C9, C19, C21, C22. (b) Asymmetry parameter 
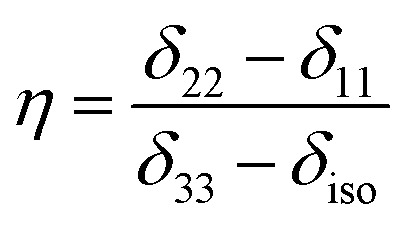
 for numerous carbon sites of folic acid.

Fig. 2 shows the ^13^C 2DPASS CP-MAS spectrum of folic acid. The direct dimension of the two-dimensional spectrum represents an infinite spinning speed spectrum and the indirect dimension represents the anisotropic spectrum. Table 1 represents the CSA parameters at crystallographically and chemically different carbon nuclei sites of folic acid. Different values of CSA parameters suggest that the molecular orientation, conformation, and electron cloud density varies significantly at different location of the molecule. For example, C19, C20, C21, C22, C23 carbon nuclei reside in glutamate moieties – but the CSA parameters are remarkably high for C20 and C23carbon nuclei compared to C19, C21, and C22. The center of gravity of the spinning CSA sideband pattern 
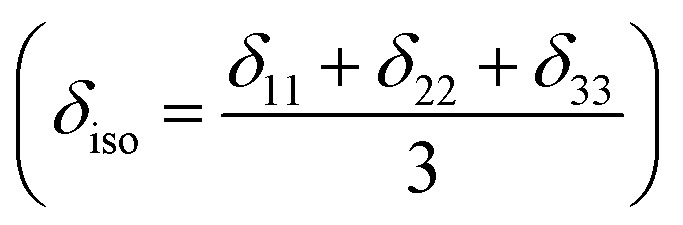
 is the isotropic chemical shift.^27–32^ Small changes in the position of the center of gravity of spinning CSA sideband pattern furnish larger changes in chemical shift anisotropy parameters.^31^ The width of the spinning CSA sideband pattern is defined as span (*Ω* = *δ*_11_ − *δ*_33_). The anisotropy (Δ*δ*) and asymmetry (*η*) parameters are defined as 
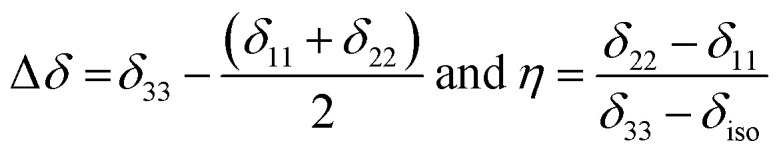
 followed by Haeberlen convention.^37^ The magnitude of anisotropy parameter says the distance of largest separation of the spinning CSA sideband pattern from the position of the center of gravity, and its sign represents on which side of the center of gravity the distance of separation is maximum. Spinning CSA sideband patterns of numerous carbon nuclei of folic acid are shown in Fig. 3. Sideband pattern is axially symmetric (*η* = 0) when *δ*_22_ is equal to *δ*_33_ or *δ*_11_. Hence, asymmetry parameter monitors the digression of the spinning CSA side band pattern from its axially symmetric shape. Table 1 says that the asymmetry parameter is greater than and equal to 0.4 for all nuclei of folic acid, and for C7, C8a, C9, C17, C19, C20, C21 and C23 carbon nuclei the value is equal or greater than 0.8, indicating that the side-band pattern is highly asymmetric for those carbon nuclei. 
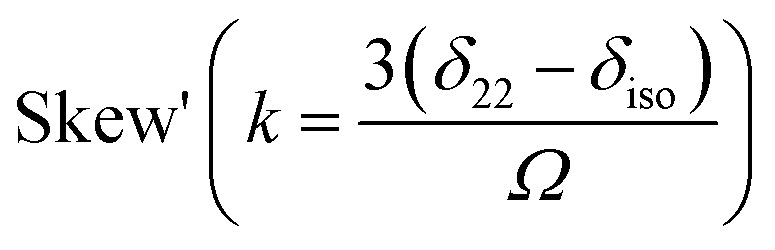
 defines the amount of orientation of the asymmetric pattern. From Fig. 3, it is clear that there is no carbon nucleus in a folic acid molecule whose spinning CSA sideband pattern is highly symmetric (*η* = 0). Fig. 4(a) and (b) portrayed vividly how anisotropy and asymmetry parameters vary at different sites of carbon nuclei. Chemical shift anisotropy parameters are remarkably high for carbon nuclei resides in heteroaromatic (C2, C4, C4a, C6, C7, C8a) and aromatic rings(C11, C12, C13, C14, C15, C16) and carbonyl group(C23, C20, C17, C4). The values of CSA parameters are comparatively lower for C9, C19, C21, and C22 carbon nuclei. For heteroaromatic and aromatic rings, the existence of π-electrons gives rise to magnetic shielding and deshielding effect. When π-electron rotates in a clockwise direction-the induced magnetic field is along the direction of the external magnetic field. As a consequence, the effective magnetic field experienced by the nucleus is increased – this is called the deshielding effect. On the other hand, the magnetic field generates by the π-electron revolves in the anticlockwise direction, is along the opposite direction of the applied magnetic field. Hence, the effective magnetic field experienced by the nucleus is decreased, this phenomenon is known as the shielding effect. As a consequence, the chemical shift anisotropy (CSA) parameters for those carbon nuclei surrounded by nonbonded electrons are substantially large.

**Table 1:** Chemical shift anisotropy parameters at numerous carbon nuclei sites of folic acid

CSA parameters of folic acid								
Carbon from different chemical environment	*δ*_11_(ppm)	*δ*_22_(ppm)	*δ*_33_(ppm)	Span (ppm)	Skew	*δ*_iso_(ppm)	Anisotropy (ppm)	Asymmetry
C23	258.7	194.8	105.8	152.9	0.2	186.4	−121	0.8
C20	250.2	181.8	114.4	135.8	0	182.1	102.1	1
C17	245	163.5	98	147	−0.1	168.9	114.2	0.9
C4	248.4	133.5	89.5	158.9	−0.4	157.1	136.9	0.5
C2	260.7	127.4	79.7	181	−0.5	155.9	157.2	0.4
C11	264.5	124.9	76.1	188.3	−0.5	155.2	164	0.4
C6	263.2	133.5	65.2	198	−0.3	154	163.8	0.6
C7	258.4	141.3	60	198	−0.2	153.2	157.7	0.8
C8a	254.3	146	58.1	196.2	−0.1	152.8	152.2	0.9
C13, C15	261.8	109.2	38.2	223.6	−0.4	136.4	188.1	0.6
C4a	241.7	100.3	44.9	196.8	−0.4	128.9	169.1	0.5
C14	219.1	103.3	41.1	178	−0.3	121.1	146.9	0.6
C16	221.1	88.5	39.9	181.2	−0.5	116.5	156.9	0.5
C12	207.5	101.1	31.3	176.2	−0.2	113.3	141.3	0.7
C19	76.3	60.2	68	38.3	0.2	58.1	−30.3	0.8
C9	62.7	48.6	36.1	26.6	0	49.2	20.3	0.9
C22	52.1	29	20.6	31.5	−0.5	33.9	27.3	0.5
C21	43.9	30.6	17.8	26	0	30.8	19.7	1

The carbonyl group does not have any axis of symmetry. Naturally, the electronic charge distribution is not spherically symmetric. By the action of electrostatic interaction, there induced polarization on the electron cloud surrounding the nucleus. This polarization affects the strength of the induced magnetic field. As a result, the strength of the magnetic field is varied in different directions.^33^ In the principal axes system (PAS), the magnetic susceptibilities (*χ*_*x*_, *χ*_*y*_, *χ*_*z*_) have different values along three mutually perpendicular directions. The McConnell equation of magnetic anisotropy for carbonyl group is^34^

1 *δ*_anis_ = {Δ*χ*_‖_(3 cos^2^ *θ*_1_ − 1) + Δ*χ*_⊥_(3 cos^2^ *θ*_2_ − 1)}/3*R*^3^

where *θ*_1_ is the angle between the radius vector and *x*-axis. *θ*_2_ is the angle between the radius vector and *z*-axis. Where Δ*χ*_‖_ = *χ*_*z*_ − *χ*_*x*_ and Δ*χ*_⊥_ = *χ*_*y*_ − *χ*_*x*_ represent anisotropic susceptibility parallel and perpendicular to the magnetic field respectively. This magnetic anisotropy is the source of lager values of CSA parameters for carbon nuclei reside on the carbonyl group.

The existence of hydrogen bonds between the water molecule and the oxygen atom connected with C4 carbon and N10 atoms of the pteridine group brings stability to the molecular structure.^26^ The existence of intermolecular and intramolecular hydrogen bonding in the pteridine group plays the foremost role to change molecular orientation and electron density. This is also another reason for the high values of CSA parameters of the carbon nuclei reside at the pteridine ring.

### 3.3 Spin-lattice relaxation time and molecular correlation time

The population of stationary states follows the Boltzmann distributions when the equilibrium is re-established among spin and lattice systems with the withdrawal of perturbation. As a consequence, the off-diagonal elements of the density operator are averaged out, and the diagonal elements are restored. That means all coherence transfer pathways have vanished. This is referred as spin-lattice relaxation. Spin-lattice relaxation mechanism is governed by homo-nuclear dipole–dipole coupling, hetero-nuclear dipole–dipole coupling, electric quadrupolar coupling, anisotropic chemical shift (CSA), and scalar coupling. Although for ^13^C nuclei, the heteronuclear dipole–dipole coupling and chemical shift anisotropy interactions play a fundamental role in the spin-lattice relaxation mechanism. The relaxation mechanism of nonprotonated ^13^C nuclei is predominated by CSA interaction. For proton relaxation, the contribution of CSA is negligible.

**Fig. 5:**
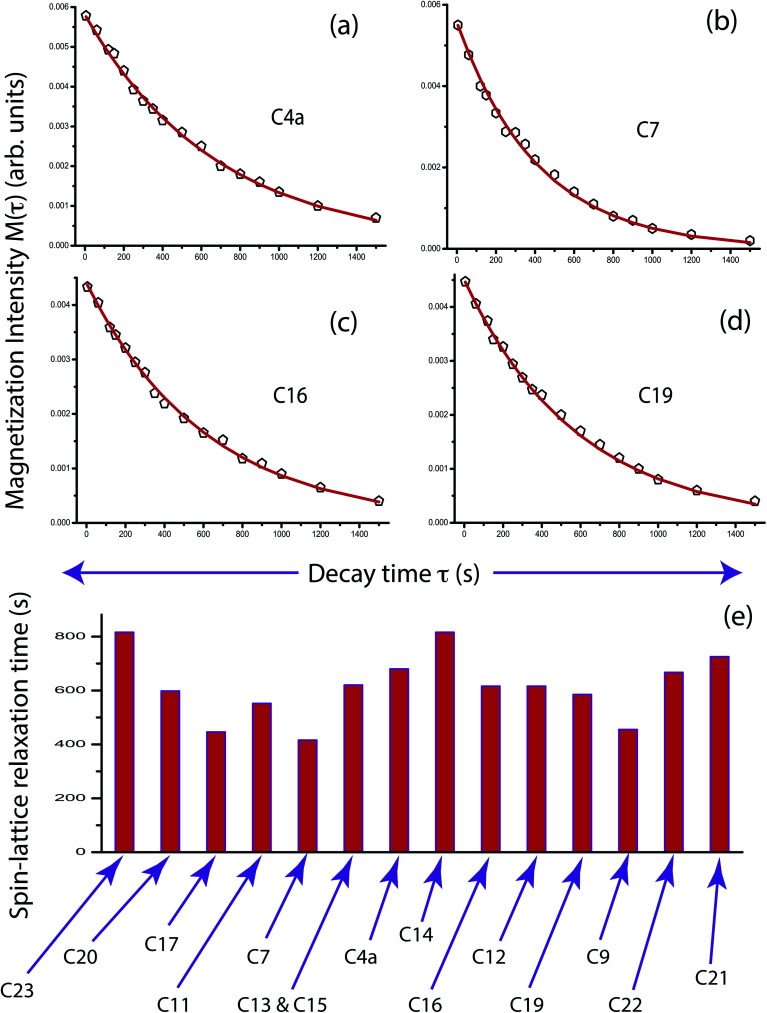
^13^C longitudinal magnetization decay curves of folic acid at (a) C4a, (b) C7, (c) C16 and (d) C19 carbon nuclei sites. (e) Bar-diagram of the spin-lattice relaxation time of carbon nuclei at numerous carbon sites of folic acid.

**Fig. 6:**
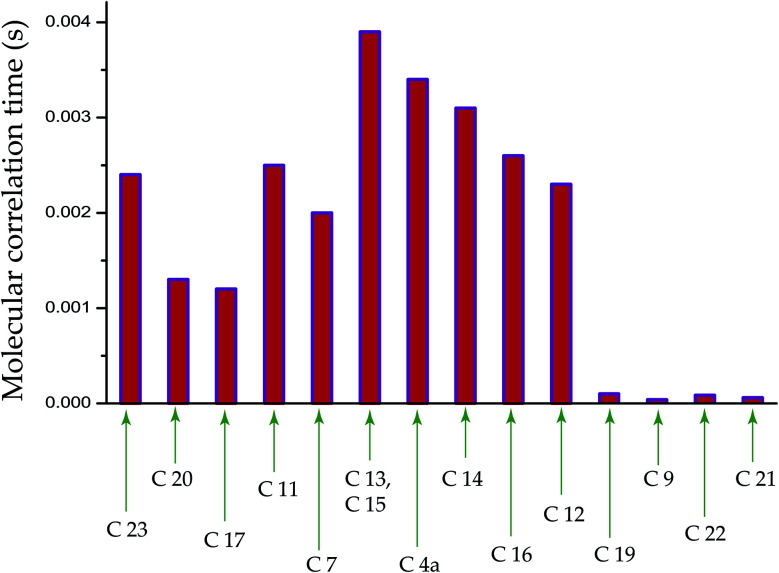
Bar-diagram of molecular correlation time of folic acid at numerous carbon sites. It is calculated by considering that the spin-lattice relaxation mechanism of ^13^C carbon nucleus is mainly governed by chemical shift anisotropy interaction and heteronuclear dipole–dipole coupling.

Fig. 5 shows ^13^C spin-lattice relaxation decay curves of folic acid at (a) C4a, (b) C7, (c) C16, and (d) C19 carbon nuclei sites. Fig. 5(e) bar-diagram and Table 2 say that spin-lattice relaxation time at numerous carbon nuclei sites of folic acid is very high and it varies from 816 s to 416 s. This significant variation of the spin-lattice relaxation time indicates that the motional degrees of freedom are different at various portions of the molecule and the motion of different molecular moieties is independent of each other. For C23 and C14 the value of spin-lattice relaxation time exceeds 800 s. Each molecule of folic acid is bonded with two other symmetry-related folic-acid molecules and water molecules by strong hydrogen bonding. Two pairs of hydrogen bonding are observed in folic acid. One pair is formed by hydrogen atom connected with N4 with N1 atom and with the alpha carboxyl group of the glutamic acid end of another symmetry-related folic acid molecule.^26^ Another pair of hydrogen bonds is fabricated between the hydrogen atom connected with N3 and the oxygen atom connected to C4 of the pteridine ring and the gamma carboxyl group of the glutamic acid end of a second symmetry-related folic acid. The second symmetry-related folic acid molecule resides in such a way that the position of the *p*-aminobenzoyl group is just below the pteridine ring of the first one. The separation between these two parallel rings of two folic acid molecule is 3.28 Å.^26^ These types of stacking arrangement of pteridine and phenyl rings and the presence of two pairs of hydrogen bonding make folic acid a robust molecule. These may be the reason for the high value of the spin-lattice relaxation time of the carbon nuclei of folic acid. The molecular correlation time (Table 4 and Fig. 6) is of the order of 10^−3^ s for most of the carbon nuclei, except C9, C19, C21, C22.

**Table 2:** Values of spin-lattice relaxation time at numerous carbon nuclei sites

Carbon from different chemical environment	Spin-lattice relaxation time (s)	Carbon from different chemical environment	Spin-lattice relaxation time (s)
C23	816 ± 20	C20	598 ± 20
C17	446 ± 10	C11	552 ± 20
C7	416 ± 10	C13 and C15	620 ± 20
C4a	680 ± 20	C14	816 ± 20
C16	616 ± 20	C12	616 ± 20
C19	585 ± 20	C9	455 ± 10
C22	667 ± 20	C21	725 ± 20

The delocalized π-electrons of the pteridine heteroaromatic ring of one molecule interact significantly with the *p*-aminobenzoyl rings of the other molecule. The stacking interaction between two molecules plays an important role in the binding of folates at the enzyme dihydrofolate reductase (DHFR) active site. If it is possible to develop folic acid antagonists, which can exploit the difference between the DHFR enzyme of normal and tumour cells, then it would be used as a valuable drug in cancer treatment.^26^ For developing such antagonists, it is necessary to have information about the structure and dynamics of folic acid in atomic scale resolution.

The local magnetic field experienced by the same nuclei situated in different chemical environments is different. The local magnetic field, experienced ^13^C nuclei, is anisotropic in nature. It fluctuates with the reorientation of the molecule with respect to the applied magnetic field, and the fluctuating local magnetic field is the source of spin-lattice relaxation. Naturally, the modification of the electronic environments with the fluctuation of the local magnetic fields can be monitored by measuring CSA parameters. CSA interaction plays a significant role in the relaxation mechanism for nuclei associated with a large value of chemical shift like carbon, nitrogen, and phosphorous for the biological molecule. The function of chemical shift anisotropy interaction in spin-lattice relaxation mechanism is expressed as^28–31^2
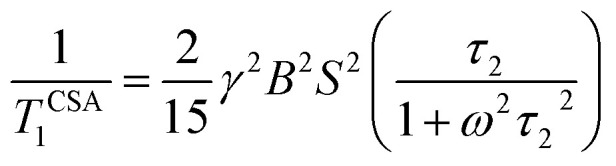
where correlation time *τ*_c_ = 3*τ*_2_ and *B* is the applied magnetic field. Where 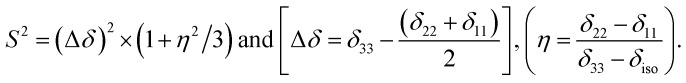


The function of heteronuclear dipole–dipole coupling on spin-lattice relaxation mechanism is expressed as^31^3
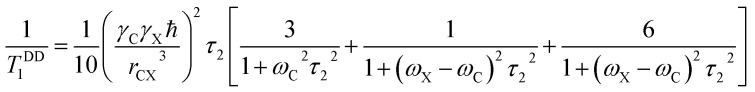
By keeping the first term only,4
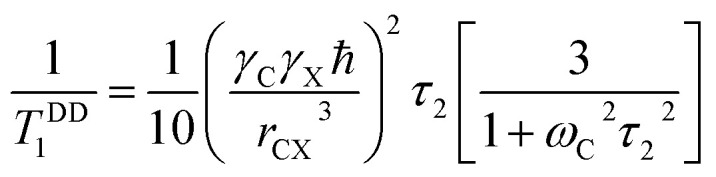
where X represent hydrogen, oxygen and nitrogen. *r*_CX_is the distance between carbon and neighbouring atoms hydrogen, oxygen, nitrogen as shown in Table 3. Larmour precession frequency *ω* = 2π*f* = 2 × 3.14 × 125.758 MHz = 789.76024 MHz; *B* = 11.74 T, *γ*_C_ = 10.7084 MHz T^−1^, *γ*_H_ = 42.577 MHz T^−1^, *ħ* = 1.054 × 10^−34^ Js.

**Table 3:** Bond-distance of folic acid

Bond	Bond distance	Bond	Bond distance
N1–C2	1.304	C22–C23	1.511
N1–C8a	1.371	C2–N24	1.368
C2–N3	1.378	N24–H31	1.009
N3–C4	1.408	N24–H32	1.011
C4–C4a	1.475	N3–H33	1.014
C4a-C8a	1.417	C4–O25	1.219
C4a-N5	1.343	C7–H34	1.090
N5–C6	1.323	C9–H35	1.102
C6–C7	1.417	C9–H36	1.104
C7–C8	1.321	N10–H37	1.013
N8–C8a	1.356	C12–H38	1.085
C6–C9	1.514	C13–H39	1.085
C9–N10	1.438	C15–H40	1.086
N10–C11	1.373	C16–H41	1.087
C11–C12	1.413	C17–O26	1.234
C12–C13	1.390	N18–H42	1.012
C13–C14	1.402	C19–H43	1.098
C14–C15	1.409	C20–O27	1.215
C15–C16	1.386	C20–O28	1.350
C16–C11	1.415	O28–H46	0.973
C14–C17	1.491	C21–H44	1.093
C17–N18	1.377	C22–H44	1.096
N18–C19	1.453	C22–H45	1.098
C19–C20	1.520	C23–O30	1.213
C19–C21	1.549	C23–O29	1.360
C21–C22	1.529	O29–H47	0.972

The spin-lattice relaxation rate for ^13^C can be written as5
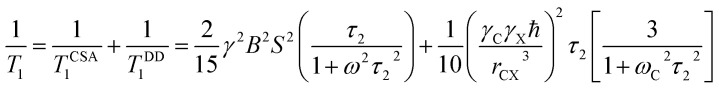


Molecular correlation time at numerous carbon nuclei sites of folic acid can be calculated by using this expression.

The bar-diagram of molecular correlation time at numerous carbon sites of folic acid is shown in Fig. 6 and Table 4. For C9, C21, and C22, molecular correlation time is of the order of 10^−5^ s. For C19 it is 10^−4^ s, but for the rest of the nuclei sites of folic acid, the molecular correlation time is of the order of 10^−3^ s. This indicates that the molecular motion is associated with different degrees of freedom.

**Table 4:** Molecular correlation time at chemically different carbon sites of folic acid

Carbon nuclei	Molecular correlation time(s)	Carbon nuclei	Molecular correlation time(s)
C23	2.4 × 10^−3^	C20	1.3 × 10^−3^
C17	1.2 × 10^−3^	C11	2.5 × 10^−3^
C7	2 × 10^−3^	C13, C15	3.9 × 10^−3^
C4a	3.4 × 10^−3^	C14	3.1 × 10^−3^
C16	2.6 × 10^−3^	C12	2.3 × 10^−3^
C19	1 × 10^−4^	C9	3.8 × 10^−5^
C22	8.6 × 10^−5^	C21	6 × 10^−5^

### 3.4 Structural dynamics of folic acid and its interaction with the receptor

**Fig. 7:**
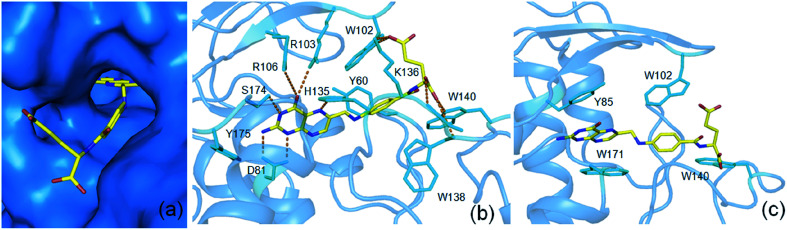
Surface representation (a) and important amino acids of folate receptor for hydrogen bond (b) and π–π stacking interactions (c) for interactions with folic acid (pdb id 4LRH)

The present study by using ^13^C 2DPASS CP-MAS NMR experiment and Torchia CP experiment the chemical shift anisotropy parameters, spin-lattice relaxation time as well as molecular correlation time at different nuclei were calculated for folic acid, whose deficiency is linked to diseases like fetal neural tube defects, cardiovascular disease, and cancers *etc.* These parameters help us to get a picture of the structure and dynamics of the molecule. It will be very interesting if similar dynamics are also present in folic acid in its interaction with the receptor in the binding pocket (Fig. 7(a)). The heteroaromatic (C2, C4, C4a, C6, C7, C8a) carbon nuclei show high CSA parameters whereas C9, C19, C21, and C22 carbon nuclei show comparatively lower values of CSA parameters as mentioned previously. The reason for the large values of the CSA parameter of these carbon nuclei of the heteroaromatic pteridine ring is due to the presence of π electrons as well as the existence of hydrogen bond interactions between C4 carbon and N10 atom. Interestingly, in the receptor bound conformation, the pteridine ring of folic acid forms hydrogen bond interactions with amino acids D81, R103, R106, H135, S174 as shown in Fig. 7(b). The spin-lattice relaxation time at numerous carbon nuclei sites varies from 816 s to 416 s and showed different motional degrees of freedom at different parts of the molecule. The crystal structure of folic acid points out the involvement of the stacking arrangement of the pteridine and phenyl rings, and the presence of two pairs of hydrogen bonding at both ends of the molecule. Similar dynamics are also observed in the case of receptor bound conformation of folic acid. The one end involving the pteridine ring forms a hydrogen bond with five amino acids as mentioned before and the other end *i.e.* the glutamic acid moiety forms another hydrogen bond with W102, K136, W138 as well as W140.^36,38^ The similar kind stacking interactions involving the pteridine ring is also found in receptor bound conformations of folic acid where amino acids Y85, W171 as well as W102 and W140 are playing an important role in π–π stacking interactions with the pteridine ring and the phenyl ring of folic acid respectively (Fig. 7(c)).

## 4. Conclusion

Folic acid is a crucial nutrient required for DNA replication, growth, and development of the fetus. It is also used as a substrate for enzymatic reactions associated with amino acid synthesis and vitamin metabolism. The structure and dynamics of this dietary supplement are exploited by extracting CSA parameters, site-specific relaxation time, and molecular correlation time at various carbon nuclei sites. At crystallographically different sites of folic acid, the spin-lattice relaxation time varies from 416 to 816 s and the molecular correlation time varies from 10^−3^ to 10^−5^ s, which indicates that the motional degrees of freedom are different at the various portion of the molecule. CSA parameters of carbon nuclei reside in the pteridine ring, *p*-aminobenzoic ring are substantially high due to the magnetic shielding/deshielding effect. Besides, the presence of intermolecular and intramolecular hydrogen bonding in the pteridine group plays a vital role to change molecular orientation and electron density. This is also another reason for the high values of chemical shift anisotropy. In stacking arrangement two consecutive folic acid molecules are oriented with each other in such a way that the position of the *p*-aminobenzoyl group of the second molecule is just below the pteridine ring of the first one. Hence, the delocalized π-electrons of the pteridine hetero-aromatic ring of the first molecule interact significantly with the *p*-aminobenzoyl rings of the second molecule. This is also another reason for high values of CSA parameters of the carbon nuclei reside at pteridine ring, *p*-aminobenzoic ring. The hydrophobic interaction between two molecules plays a vital role in the binding of folic acid at the DHFR active site. CSA parameters of carbonyl group carbon are also high due to the existence of magnetic anisotropy. Elucidation of the structure and dynamics of folic acid like this way will illuminate the path of inventing advanced drugs in Pharmaceutical Industries.

## Conflicts of interest

There are no conflicts to declare.

## Supplementary Material

## References

[cit1] Greenberg J. A., Bell S. J., Guan Y., Yu Y.-h. (2011). Folic acid supplementation and pregnancy: more than just neural tube defect prevention. Reviews in Obsterics and Gynecoligy.

[cit2] Braga D., Chelazzi L., Grepioni F., Maschio L., Nanna S., Taddei P. (2016). Folic acid in the solid state: a synergistic computational, spectroscopic, and structural approach. Cryst. Growth Des..

[cit3] Dessie M. A., Zeleke E. G., Workie S. B., Berihum A. W. (2017). Folic acid usage and associated factors in the prevention of neural tube defects among pregnant women in Ethiopia: cross-sectional study. BMC Pregnancy Childbirth.

[cit4] Abyar F., Novak I. (2019). Conformational analysis and electronic structure of folic acid: a theoretical study. J. Mol. Liq..

[cit5] Wibowo A. S., Singh M., Reeder K. M., Carter J. J., Kovach A. R., Meng W., Ratnam M., Zhang F., Dann III C. E. (2013). Structures of human folate receptors reveal biological trafficking states and diversity in folate and antifolate recognition. Proc. Natl. Acad. Sci. U. S. A..

[cit6] Ramsey N. F. (1950). Magnetic Shielding of Nuclei in Molecules. Phys. Rev..

[cit7] Ramsey N. F. (1952). Chemical effects in nuclear magnetic resonance and in diamagnetic susceptibility. Phys. Rev..

[cit8] Tycko R., Dabbagh G., Mirau P. A. (1989). A. Determination of chemical shift anisotropy lineshapes in a two-dimensional magic angle spinning NMR experiment. J. Magn. Reson..

[cit9] Liu S. F., Mao J. D., Schmidt-Rohr K. (2002). A robust technique for two-dimensional separation of undistorted chemical shift anisotropy powder patterns in magic angle spinning NMR. J. Magn. Reson..

[cit10] Chan J. C. C., Tycko R. (2003). Recoupling of chemical shift anisotropies in solid state NMR under high speed magic angle spinning and in uniformly ^13^C labelled systems. J. Chem. Phys..

[cit11] Hou G., Byeon In-Ja L., Ahn J., Gronenborn A. M., Polenova T. (2012). Recoupling of chemical shift anisotropy by R-symmetry sequences in magic angle spinning NMR spectroscopy. J. Chem. Phys..

[cit12] Bax Ad, Szeverenyi N. M., Maciel G. E. (1983). Correlation of isotropic shifts and chemical shift anisotropies by two-dimensional Fourier-transform magic angle hopping NMR spectroscopy. J. Magn. Reson..

[cit13] Bax Ad, Szeverenyi N. M., Maciel G. E. (1983). Chemical shift anisotropy in powdered solids studied by 2D FT CP/MAS NMR. J. Magn. Reson..

[cit14] Bax Ad, Szeverenyi N. M., Maciel G. E. (1983). Chemical shift anisotropy in powdered solids studied by 2D FT NMR with flipping of the spinning axis. J. Magn. Reson..

[cit15] Bax Ad, Szeverenyi N. M., Maciel G. E. (1983). Correlation of isotropic shifts and chemical shift anisotropies by two-dimensional Fourier-transform magic angle hopping NMR spectroscopy. J. Magn. Reson..

[cit16] Gan Z. (1992). High-resolution chemical shift and chemical shift anisotropy correlation in solids using slow magic angle spinning. J. Am. Chem. Soc..

[cit17] Dixon W. T. (1982). Spinning-sideband-free and spinning-sideband-only NMR spectra in spinning samples. J. Chem. Phys..

[cit18] Antzutkin O. N., Shekar S. C., Levitt M. H. (1995). Two-dimensional sideband separation in magic angle spinning NMR. J. Magn. Reson., Ser. A.

[cit19] Walder B. J., Dey K. K., Kaseman D. C., Baltisberger J. H., Grandinetti P. J. (2013). Sideband separation experiments in NMR with phase incremented echo train acquisition. J. Chem. Phys..

[cit20] Ghosh M., Sadhukhan S., Dey K. K. (2019). Elucidating the internal structure and dynamics of α-chitin by 2DPASS-MAS-NMR
and spin-lattice relaxation measurements. Solid State Nucl. Magn. Reson..

[cit21] Ghosh M., Prajapati B. P., Kango N., Dey K. K. (2019). A comprehensive and comparative study of the internal structure and dynamics of natural β-keratin and regenerated β-keratin by solid state NMR spectroscopy. Solid State Nucl. Magn. Reson..

[cit22] Ghosh M., Kango N., Dey K. K. (2019). Investigation of the internal structure and dynamics of cellulose by ^13^C-NMR relaxometry and 2DPASS-MAS-NMR measurements. J. Biomol. NMR.

[cit23] Dey K. K., Ghosh M. (2020). Understanding the effect of deacetylation on chitin by measuring chemical shift anisotropy tensor and spin lattice relaxation time. Chem. Phys. Lett..

[cit24] Dey K. K., Gayen S., Ghosh M. (2019). Investigation of the detailed internal structure and dynamics of itraconazole by solid-state NMR measurements. ACS Omega.

[cit25] Dey K. K., Gayen S., Ghosh M. (2020). Understanding the correlation between structure and dynamics of clocortolone pivalate by solid state NMR measurement. RSC Adv..

[cit26] Mastropaolo D., Camerman A., Camerman N. (1980). Folic acid: crystal structure and implications for enzyme binding. Science.

[cit27] Herzfeld J., Berger A. E. (1980). Sideband intensities in NMR spectra of samples spinning at the magic angle. J. Chem. Phys..

[cit28] Nicholas M. P., Eryilmaz E., Ferrage F., Cowburn D., Ghose R. (2010). Nuclear spin relaxation in isotropic and anisotropic media. Prog. Nucl. Magn. Reson. Spectrosc..

[cit29] Orendt A. M., Facelli J. C. (2007). Solid state effects on NMR chemical shifts. Annu. Rep. NMR Spectrosc..

[cit30] Tjandra N., Szabo A., Bax Ad. (1996). Protein backbone dynamics and 15B chemical shift anisotropy from quantitative measurement of relaxation interference effects. J. Am. Chem. Soc..

[cit31] Dais P., Spyros A. (1995). ^13^C nuclear magnetic relaxation and local dynamics of synthetic polymers in dilute solution and in the bulk state. Prog. Nucl. Magn. Reson. Spectrosc..

[cit32] Anet F. A. L., O'Leary D. J. (1992). The shielding tensor Part II: Understanding its strange effect on relaxation. Concepts Magn. Reson..

[cit33] Abraham R. J., Mobli M., Smith R. J. (2003). 1H chemical shifts in NMR: Part 19† Carbonyl anisotropies and steric effects in aromaticaldehydes and ketones. Magn. Reson. Chem..

[cit34] McConnell H. M. (1957). Theory of Nuclear Magnetic Shielding in Molecules: Long-Range Dipolar Shielding of protons. J. Chem. Phys..

[cit35] Torchia D. A. (1978). The measurement of proton-enhanced carbon-13 T1 values by method which suppresses artifacts. J. Magn. Reson..

[cit36] Chen C., Ke J., Zhou X. E., Yi W., Brunzelle J. S., Li J., Yong Eu-L., Eric Xu H., Melcher K. (2013). Structural basis for molecular recognition of folic acid by folate receptors. Nature.

[cit37] Haeberlen U. , High Resolution NMR in Solids: Selective Averaging, Academic Press, New York, 1976

[cit38] Sahoo B. R., Maharana J., Patra M. C., Bhoi G. K., Lenka S. K., Dubey P. K., Goyel S., Dehury B., Pradhan S. K. (2014). Structural and dynamic investigation of bovine folate receptor alpha(FOLR1), and role of ultra-high temperature processing on conformational and thermodynamic characteristics of FOLR1-folate complex. Colloids Surf., B.

